# Photoaligning Polymeric Command Surfaces: Bind, or Mix?

**DOI:** 10.3390/polym15214271

**Published:** 2023-10-30

**Authors:** Ameer R. K. Nassrah, Marianna Batkova, Natália Tomašovičová, Tibor Tóth-Katona

**Affiliations:** 1 Institute for Solid State Physics and Optics, Wigner Research Centre for Physics, P.O. Box 49, H-1525 Budapest, Hungary; nassrah.ameer@wigner.hu; 2 Faculty of Science, Eötvös Loránd University, P.O. Box 32, H-1518 Budapest, Hungary; 3 Institute of Experimental Physics, Slovak Academy of Sciences, Watsonová 47, 04001 Kosice, Slovakia; batkova@saske.sk (M.B.); nhudak@saske.sk (N.T.)

**Keywords:** nematic–polymer interface, polymer–air interface, photoalignment, optical sensors, optical actuators

## Abstract

We compare photoaligning properties of polymer layers fabricated from the same constituents: polymethyl-methacrylate (PMMA) and azo-dye Disperse Red 1 (DR1), either chemically attached to the PMMA main-chain, or physically mixed with it. Photoaligning properties depend on the preparation method drastically. Photoalignment was found to be far more efficient when PMMA is functionalized with DR1 compared to the case of physically mixing the constituents. This finding is supported by atomic force microscope (AFM) scans monitoring the light-induced changes at the polymer–air interface, and revealing a photoinduced mass transfer, especially in the case of functionalized PMMA.

## 1. Introduction

Photoalignment of liquid crystals (LCs) [1,2,3]—discovered more than three decades ago [4,5,6,7]—can be achieved in contactless manner by proper light irradiation. It provides an alternative to standard aligning methods (such as mechanical rubbing of polyimide layers), by which, in many cases, the drawbacks and limitations of the rubbing method can be avoided. For example, in contrast to photoalignment, mechanical rubbing may produce and accumulate static charges and dust particles, can damage the alignment layer, cannot align LCs in enclosed areas, or in microfluidic channels, etc. Since in these photoaligning systems, a very small number of photochromic derivatives anchored on a substrate commands the alignment of a very large number of LC molecules in contact with it, the surfaces were referred to as “command surfaces” [4].

The above-described advantages, combined with the relative ease of patterned photoalignment make the application potential of photoalignment very high in display and photonic devices [8]. Those applications include but are not limited to the fabrication of novel displays, optically rewritable flexible e-paper, tunable LC lenses, LC sensors, optical waveguides, patterned polarizers, phase retarders, optical filters, optical gratings, etc. [8,9,10,11]. Many of these devices cannot be achieved by other standard aligning methods.

Command surfaces are usually produced as monolayers of dye derivatives anchored on a substrate [4,12,13,14,15,16], or in the form of dye derivatives embedded in polymers and spin-coated on the substrate. In the latter case, prior to the spin-coating, dye derivatives can be either physically mixed with (doped to) the polymer [5,7,17,18,19,20], or chemically attached to the polymer chain (in most cases by covalent bonding) [21,22,23,24,25,26,27,28,29,30,31,32,33,34,35,36].

In the above-cited works, azobenzene dye derivatives—exhibiting trans-cis (*E*/*Z*) isomerization [37]—were used as photochromic units, due to their remarkable photo- and chemical stability, relative ease of synthesis, good solubility in liquid crystals, and due to the reversibility of their polarization dependent photoisomerization. Namely, the polarized irradiation selectively excites the trans azo isomers depending on their orientation, and rapid successive trans-cis-trans isomerization cycles result in the orientation of the azobenzene long axis perpendicular to the light polarization [37]. Many azobenzene derivatives exhibit liquid crystalline properties themselves. Nonetheless, other photochromic units, as well as other photoreactions are also exploited to implement photoaligning command surfaces, and are nicely summarized, e.g., in Table 2 of Ref. [38]. As for the polymer host, in the case of physical mixing with azobenzene derivatives, polyimide [5,7,18,19,20] and polyvinyl alcohol [17] was used. On the other hand, various azo-monomers were chemically attached to the modified main-chain of polyacrylates [33,34], polymethacrylates [24,25,29,30,31,32], polyamides [35], polyvinyl alcohols [21,22,23,26], or polysiloxanes [27,28].

The composition variety of the command surfaces in the above-mentioned photoaligning systems largely prevents direct comparison of the benefits and disadvantages of the two fabrication methods (physical mixing/doping, and chemical binding). Moreover, our recent investigations [39,40,41] have shown that the mechanism and the efficiency of photoalignment/photocontrol do not depend on the composition of the polymer layer exclusively, but also on the liquid crystal material in contact with the command surface. Therefore, for comparative investigations on the two systems, the use of the same polymer, same photochromic unit, and same liquid crystal compound is desirable. To the best of our knowledge, such a comparative investigation has not been reported yet, and one of the aims of the present work is to fill this gap. Nonetheless, the comparison of the photoalignment on command surfaces having disparate chemical compositions allows us to notice some general trends. It seems that command surfaces prepared by physically mixing the polymer with the photochromic units require significantly higher light power for photoalignment. The dynamics of the photoalignment is slower, and the photoalignment angle is smaller, when compared to the command surfaces made of polymers grafted with photochromic units—see, e.g., in Ref. [42].

The second aim of the present work regards the investigations on the photoinduced mass transfer [43,44]. Namely, when the layer of a polymer grafted with azobenzene derivative is illuminated with a sinusoidal intensity pattern (obtained from the interference of two coherent laser beams), a large-scale modulation of the free surface is obtained [45,46,47], which is referred to as surface relief gratings (SRG). The amplitude of the modulations was found in the order of 100nm, and the periodicity of the grooves matched the periodicity of the illuminating interference pattern. Similarly, when the surface of the azobenzene-containing polymer film is irradiated with a single Gaussian laser beam with a radius focused down to few μm, a crater is formed, due to the photoinduced mass transfer [48,49]. Although the mechanism with which the molecular trans-cis-trans cyclic photoisomerization converts to a macroscopic mass transfer is still under debate, the dependence of the created surface reliefs on the spatial modulation of the light intensity and on the light polarization is known [44].

In the present work, we do not produce an interference pattern resulting in SRG, nor do we focus the Gaussian laser beam to create a crater with a radius of the order of μm, but rather we expand the laser beam to obtain photoalignment over a considerably large area having a diameter of few millimeters. However, the question of how the photoaligning illumination modifies the free surface of the polymer and whether a photoinduced mass transfer is detectable under these conditions is still relevant.

As a final remark, we note here that SRG formation has already been investigated on the same systems [polymethyl-methacrylates (PMMA) doped-, or functionalized with Disperse Red 1 (DR1) dye] [50], as we consider in the present work. The formation of SRG was found in both the DR1-doped PMMA system and in the DR1-functionalized polymer system, but the surface modulation was an order of magnitude larger in the latter case. Moreover, the surface grating effect was permanent only in the functionalized system.

## 2. Materials and Methods

**Figure 1:**
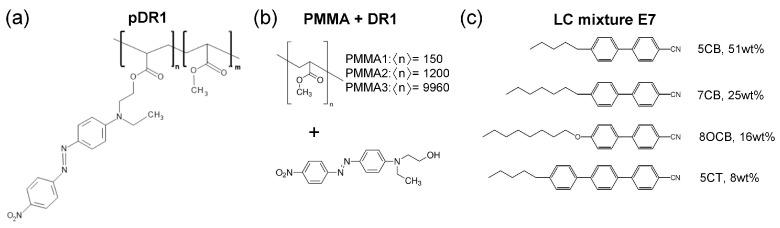
Molecular structures of (**a**) photosensitive polymer pDR1, n:m≈1:9, (**b**) polymethyl-methacrylates (PMMA) of different number-average degree of polymerization 〈n〉 physically mixed the azo-dye Disperse Red 1 (DR1), and (**c**) the nematic liquid crystal mixture E7.

For the experiments on photoalignment, liquid crystal cells of typical thickness d∼10μm were prepared from a reference and a photosensitive plate, into which the nematic liquid crystal mixture E7 was filled by capillary action. The composition of the E7 mixture is shown in Figure 1c and has the nematic-to-isotropic phase transition temperature at $T_{NI}=60\;{}^{\circ }C$. For the reference plate rubbed polyimide slide was used from E.H.C. Co. (Tokyo, Japan), which ensures a fixed, planar orientation of the liquid crystal at the surface (i.e., the director **n** is parallel with the surface of the plate). The photosensitive plates were prepared by spin-coating on the glass substrate either the 2wt% solution of polymethyl-methacrylate (PMMA) functionalized with Disperse Red 1 (DR1) dye [pDR1, Figure 1a], or the 2wt% solution of the mixture PMMA+DR1 [Figure 1b] in toluene. Prior to the spin-coating, the glass substrates were cleaned by sonication, following the recipe of Ref. [51]: for 10 min in each of the following solvents in the order of ethanol, trichloroethylene, methylene chloride, ethanol again, rinsed by Millipore water (obtained by ELGA Purelab Option), and dried with a nitrogen jet. Spin-coating was performed at 800 rpm for 5 s, and then at 3000 rpm for 30 s (all with spin acceleration of ±1000 rpm/s). The spin-coated substrates were baked in an oven for about 2 h at $140\;{}^{\circ }C$. The thickness of the polymer layer was estimated to be of the order of 0.1μm, based on the spin-coating experiments on PMMA [51]. The reference and the photosensitive plates were assembled with spacers, and the thickness of the assembled cells was measured using the interferometric method. Prior to and during filling the cell with the LC mixture E7, it was illuminated with light polarized perpendicular to the rubbing direction on the reference plate.

Mixtures of PMMA+DR1 were prepared with various PMMA polymers having different number-average degrees of polymerization from 〈n〉=150 to 〈n〉=9960 as indicated in Figure 1b, all obtained from Sigma-Aldrich and used as received. Namely, on one hand, the increase in the degree of polymerization increases the glass transition temperature ($T_{g}$) of PMMA [52,53]. On the other hand, $T_{g}$ in thin polymer films is known to influence the dynamic processes of other contacting materials, thus the difference in $T_{g}$ of the underlying polymer may substantially affect the photoalignment behavior of the layer [54]. For the midpoint of the glass transition temperature, values of $T_{g}=105\;{}^{\circ }C$ and $T_{g}=125{\;}^{\circ }C$ were given by the provider for PMMA1 and PMMA3, respectively [see Figure 1b], while for pDR1 [Figure 1a] a $T_{g}=119{\;}^{\circ }C$ was measured [55]. In the photoalignment experiments, the content of DR1 in the mixture with PMMA has also been varied in a wide range: from 6.2wt% of DR1 up to 42wt% of DR1. The former concentration corresponds to the DR1 content of pDR1.

The choice of the LC mixture E7 for further measurements on photoalignment is based on our previous studies. Namely, besides the conveniently wide temperature range of the nematic liquid crystal phase (up to $T_{NI}=60{\;}^{\circ }C$), at the interface with the pDR1 polymer layer, E7 showed the richest variety of photo-induced mechanisms compared to other nematic LC compounds [40,41]. At lower temperatures (close to room temperature), an almost complete azimuthal photoalignment is observable, achieved by a twist deformation, which relaxes back relatively fast upon switching off the exciting irradiation. In contrast, at high temperatures (close to $T_{NI}$) the azimuthal photoalignment vanishes, and instead, besides a temperature-induced anchoring transition, zenithal photoalignment occurs [39]. We have attributed this complex behavior to the different temperature dependence of the azimuthal and zenithal anchoring strengths [39], and to the molecular structure of the rigid core of E7 components that contain biphenyl, capable of establishing π-π aromatic interaction with the azo-benzene of pDR1 [40].

The pump-probe optical setup, combined with lock-in technique for the photoalignment measurements, as well as the methods for the determination of the azimuthal (in-plane) photoalignment angle, φ, and for the detection of the zenithal (out-of-plane) photoalignment were described in details in Ref. [41]. The only difference between the measurement method presented here and those reported in Refs. [39,40,41] is that for the determination of the azimuthal photoalignment angle, here, the polarization of the pump beam enclosed $45^{\circ }$ with the initial director orientation **n** (instead of being parallel with it as in Refs. [39,40,41]). The reason for this change was to avoid the accidental creation of a supertwist deformation in the LC cell [56]. Consequently, when switched on, the pump beam is expected to induce twist deformation in the LC cell with $\varphi =45^{\circ }$ at the photosensitive plate for the complete azimuthal photoalignment.

Atomic force microscopy (AFM) scans on the photosensitive substrates prior to and after the polarized laser illumination were carried out with an Agilent 5500 AFM system equipped with PicoView 1.14.3 control software. The images were acquired in the semi-contact (tapping) mode using medium soft silicon cantilevers (Oxford Instruments, Abingdon, Oxfordshire, England, model AC240TS-R3) with the resonant frequency of 70 kHz (typ.), and spring constant of 2 N/m (typ.). The measurements were performed at ambient relative humidity of 30–40% at room temperature. The captured images were processed using freely available software Gwyddion 2.63, (Free Software Foundation, Inc., 51 Franklin Street, Boston, MA, USA) [57].

## 3. Results

### 3.1. Photoalignment Measurements

**Figure 2:**
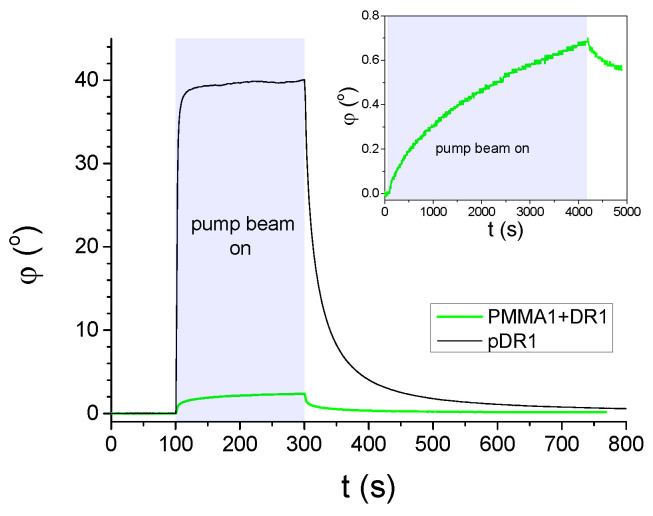
Azimuthal photo-reorientation angle φ in time, measured at temperature $T_{NI}-T=32{\;}^{\circ }C$ in cells filled with E7 and having pDR1, or PMMA1+DR1 mixture (with 30wt% DR1 content) layer as a photosensitive plate. The pump-beam was switched on at t=100s, and switched off at t=300s. The power of the pump beam was P=10mW in the case of the cell with pDR1, and P=70mW for the cell with PMMA1+DR1. Inset: long time illumination measurement on another location of the cell with PMMA1+DR1 (pump-beam switched on at t=100s, and switched off at t=4200s).

In Figure 2 results on the azimuthal photoalignment/photoreorientation are shown, measured on LC cells with a photosensitive polymer layer from pDR1, as well as from PMMA1+DR1 mixture (with 30wt% DR1 content) at a temperature $T_{NI}-T=32{\;}^{\circ }C$, close to the room temperature. As we mentioned in Section 2, the polarization of the pump beam encloses $45^{\circ }$ with the initial director orientation **n** at the reference plate.

Results on the azimuthal photoalignment in the LC cell with pDR1 are in agreement with the previous results [39,40]: at low temperatures, upon light excitation, the azimuthal photoreorientation angle at the surface of pDR1 reaches a saturated value of $\varphi_{sat}\approx 40^{\circ }$ relatively fast, i.e., almost a complete azimuthal photoalignment occurs via photo-induced twist deformation in the LC layer. When the photo-excitation is switched off, a relatively fast back-relaxation is observed—the system returns to the initial, planar orientation.

In contrast, in the LC cell with the polymer layer from the PMMA1+DR1 mixture, upon the light excitation (despite the much higher light power), a much smaller change in the azimuthal photoalignment angle was observed (typically up to $\varphi \approx 3^{\circ }$), and the small photo-induced change does not seem to saturate even for long illumination times—see the inset of Figure 2. When the pump beam is switched off, the small photo-induced change in φ relaxes back slowly.

**Figure 3:**
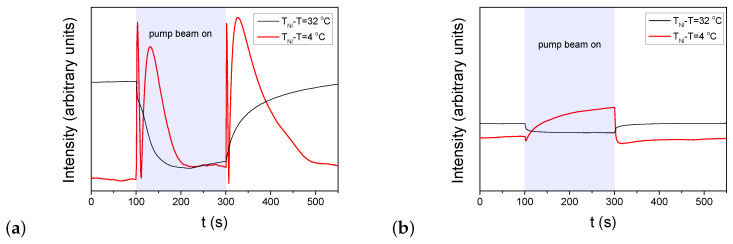
Temporal variation of the transmitted light intensity of the probe beam measured in E7 cells at two different temperatures in the setup for detection of zenithal photo-reorientation (pump beam polarization perpendicular to **n**, probe beam polarization encloses $45^{\circ }$ with **n**—for the details of experimental setup refer to [41]). The pump-beam of power *P* switched on at t=100s and switched off at t=300s. (**a**) pDR1, P=10mW; (**b**) PMMA1+DR1 mixture (with 30wt% DR1 content), P=70mW.

For the investigations on the zenithal photoalignment, the polarization of the probe beam has enclosed $45^{\circ }$ with **n** (to maximize the sensitivity), while the polarization of the pump beam was set perpendicular to **n**, which ensures the absence of the azimuthal photoalignment and its influence on the results. When significant zenithal photoalignment occurs, due to the change in the birefingence, oscillations appear in the transmitted light intensity of the probe beam when the pump beam is switched on/off. The results on zenithal photoalignment are shown in Figure 3 for two (low and high) temperatures and for both cells with pDR1 and PMMA1+DR1.

Again, results on the zenithal photoalignment in the LC cell with pDR1 [Figure 3a] are in agreement with the previous results [39]: at low temperatures, upon irradiation, only a slight change in the transmitted light intensity was observed, indicating the absence of zenithal photoalignment; at high temperatures, however, oscillations in the transmitted light intensity, both when pump beam is switched on and off, clearly indicate a significant zenithal photoalignment. In the analysis of Ref. [39], the zenithal photoalignment angle was estimated to be in the range between $\theta_{photo}=33^{\circ }$ and $\theta_{photo}=42.5^{\circ }$.

In contrast, in the LC cell with the polymer layer from the PMMA1+DR1 mixture, upon the light excitation (despite the much higher light power), only a slight change in the transmitted light intensity of the probe beam was observed in the whole temperature range (from room temperature, up to $T_{NI}$)—see Figure 3b. This slight change at all temperatures may originate either from a small misalignment of the director at the two bounding surfaces, or from a small misalignment of the polarization direction of the pump beam and **n**, or eventually, from a slight zenithal photoalignment as it was discussed in Ref. [39].

We note here, that in the photoalignment measurements presented above, only the results on the mixture of PMMA1 [with the smallest 〈n〉—see Figure 1b] with 30wt% DR1 are shown. Other polymer layers have also been prepared from the mixtures of PMMA1, PMMA2, or PMMA3 with DR1 in different concentrations (with DR1 content ranging from 6.2wt% to 42wt%). Photoalignment measurements on LC cells with these substrates have led to essentially the same results as those presented in Figure 2 and Figure 3b. Therefore, it seems that the photoaligning properties of polymer layers composed of the PMMA and DR1 mixture do not depend substantially on the degree of polymerization, nor on the concentration of the photochromic material.

### 3.2. Atomic Force Microscopy (AFM) on Photosensitive Substrates

Previous investigations [40,41] have shown that at the pDR1 surface, the relatively fast back-relaxation of the azimuthal photoalignment angle φ when the exciting illumination is switched off (as shown in Figure 2), happens only when the pDR1 surface interfaces LC material having biphenyl in its rigid core. When pDR1 is in contact with LCs not having biphenyl in the molecular structure, there is no back-relaxation, or it is extremely slow. Therefore, it is reasonable to expect, that the pDR1 surface in contact with the air will not relax back on the time scales of hours after the exciting illumination. This assumption was a prerequisite for the AFM measurements presented in this subsection.

Glass substrates with pDR1 and with PMMA1+DR1 mixture (having 35wt% DR1 content) were prepared identically as those for photoalignment measurements. AFM scans were performed at certain (identical) locations prior and after the illumination with polarized light having intensity of the order of $100\;\mathrm{mW}/{\mathrm{cm}}^{2}$ (at λ=405nm).

**Figure 4:**
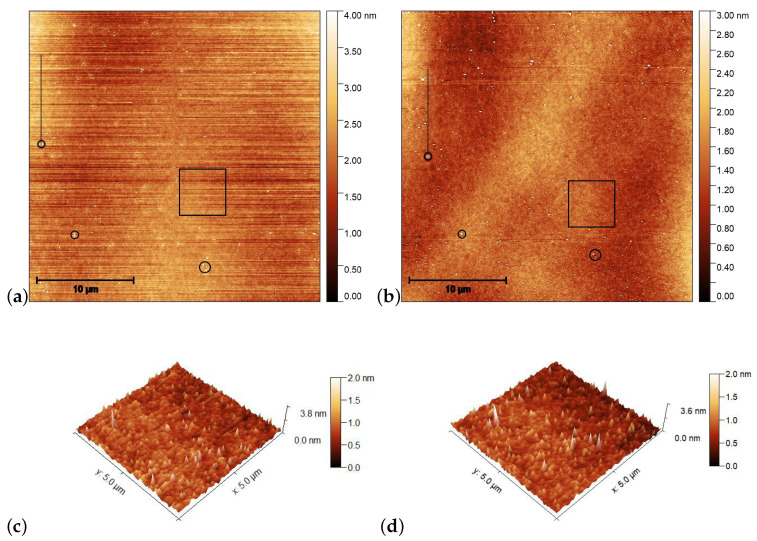
AFM images taken on the pDR1 surface prior [(**a**,**c**)], and after [(**b**,**d**)] the laser illumination. Encircled spots in (**a**,**b**) serve as reference locations to identify the same area for AFM scanning prior and after the illumination, while the squares denote the area that are presented in (**c**,**d**) in three dimensions. The vertical lines in (**a**,**c**) denote the locations from which the one-dimensional profiles are taken (to be discussed later).

AFM scans on the pDR1 substrate are shown in Figure 4 prior [(a) and (c)], and after [(b) and (d)] the illumination. The polarization direction of the illumination was set vertically (along the y direction).

**Figure 5:**
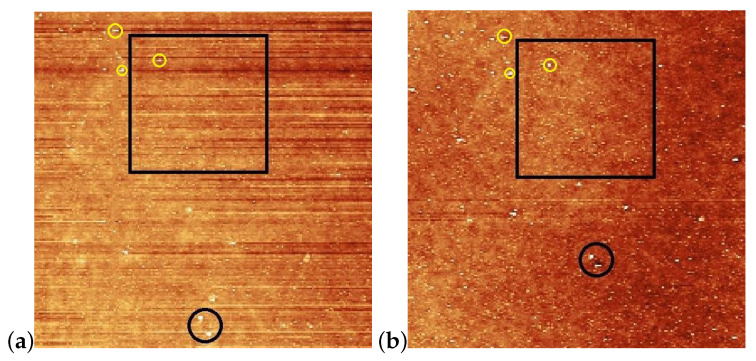
Enlarged images of the neighborhood of squares shown in Figure 4a,b with the new reference locations encircled with yellow; (**a**) prior the laser illumination; (**b**) after the illumination.

For the determination of the influence of the illumination, it is critical to compare the same area of the substrate before and after the irradiation. For that purpose, reference locations were selected which can be undoubtedly identified both prior to and after the irradiation. Those locations are marked with black circles in Figure 4a,b. One can see some photoinduced changes immediately from the AFM scans. First, changes in the surface relief: horizontal grooves (along the x-axis) present prior to the illumination (presumably caused by the spin-coating) [Figure 4a,c], disappear after the illumination [Figure 4b,d]. The relative positions of the reference locations have slightly changed—*cf*. the positions of the locations marked with black circles in Figure 4a,b. A closer look at the enlarged images shown in Figure 5 proves that even within the original reference location (black circle) the two white spots change their relative positions upon the irradiation. These are strong indications of the photoinduced mass transfer. Therefore, the selection of the area for the three-dimensional representation, shown in Figure 4c,d, was based on new reference locations, indicated by yellow circles in Figure 5a,b, that do not change their relative positions upon illumination.

**Figure 6:**
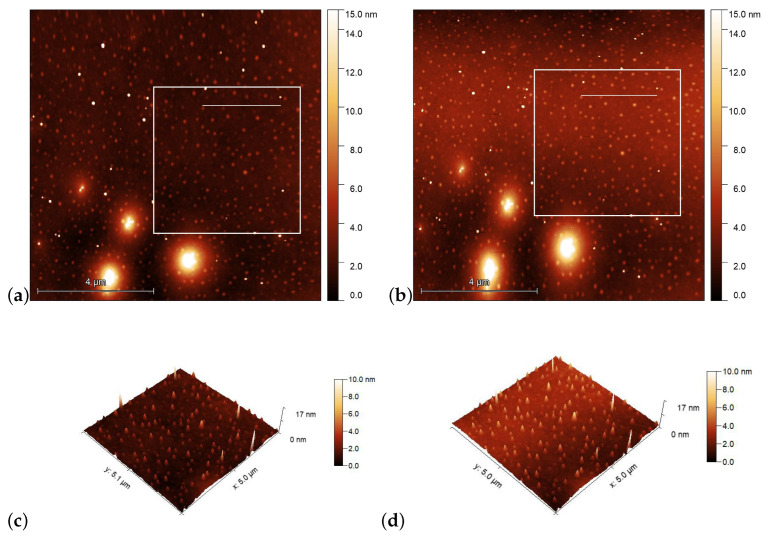
AFM images taken on the surface of PMMA1+DR1 mixture (with 35wt% DR1 content) prior [(**a**,**c**)], and after [(**b**,**d**)] the laser illumination. The squares in (**a**,**b**) denote the area that is presented in (**c**,**d**) in three dimensions, while the horizontal lines in squares denote the locations from which the one-dimensional profiles shown in Figure 7b are taken.

AFM scans on the PMMA1+DR1 substrate are shown in Figure 6 before [(a) and (c)], and after [(b) and (d)] the illumination. Again, the polarization direction of the illumination was set vertically (along the y direction). Here, no obvious photoinduced change is detectable.

**Figure 7:**
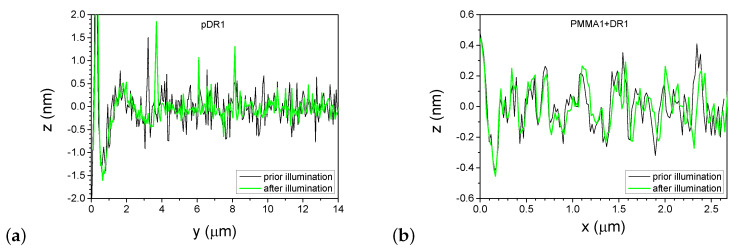
One dimensional profiles along the lines shown in Figure 4 and Figure 6, taken before and after the laser illumination; (**a**) pDR1; (**b**) PMMA1+DR1.

A more detailed information about the photoinduced changes can be obtained from one-dimensional profiles presented in Figure 7, taken along the lines shown in Figure 4 and Figure 6, before and after the laser illumination.

One-dimensional profiles taken on the pDR1 substrate along the line indicated in Figure 4a,b are shown in Figure 7a. As a starting point (y=0) for the profiles, one of the reference points—the high peak encircled in Figure 4a,b was used. Obviously, the illumination smoothens the z(y) profile: the roughness of z(y)≈±0.5nm evidently becomes smaller after the illumination. At the same time, the smoothing causes a significant lateral photoinduced mass transfer too: for example, the high peak at y=3.2μm prior to the illumination has moved to the position y=3.7μm after the illumination.

One-dimensional profiles taken on the PMMA1+DR1 substrate along the line indicated in Figure 6a,b are shown in Figure 7b. Here, the changes in the profile after the illumination are minimal and are very close to the resolution of the AFM. The peaks and valleys undergo only slight changes upon illumination and can be identified individually before and after the illumination. Note that the last double peak at x≈2.4μm shifts laterally by about 40nm upon illumination, which is more than an order of magnitude smaller shift than that observed on pDR1 substrate, indicating a very small light-induced mass transfer compared to that in pDR1.

## 4. Discussion

Photoalignment measurements on LC cells with a pDR1 substrate and with E7 nematic LC mixture have confirmed our previous results [39,40] concerning both the azimuthal and the zenithal photoalignment. In contrast to that, measurements on LC cells with various PMMA+DR1 substrates, filled with E7 have resulted in very small, but measurable, azimuthal photoalignment angles, while the zenithal photoalignment was found negligible, if it exists at all.

It is worth comparing the results of azimuthal photoalignment obtained here on PMMA+DR1 substrates, with previous studies on a different system [20,42]. In Refs. [20,42], the command surface was prepared from the mixture of polyimide (PI) and azo dye Disperse Orange 3 (DO3). More precisely, the substrates were coated with a saturated solution of DO3 in polyamic acid solution, and the iridization was completed thermally. Under similar illumination conditions, a much larger azimuthal photoalignment angle was found in cells with PI+DO3 substrate ($\varphi \geq 15^{\circ }$) than with PMMA+DR1 command surface ($\varphi \leq 3^{\circ }$). We have also prepared substrates from PMMA+DO3 mixtures and we have detected very similar photoalignment performance as with PMMA+DR1 (typically, $\varphi \leq 3^{\circ }$). Therefore, one can assume that the poor photoalignment efficiency originates from the PMMA matrix.

For the very weak photo-response measured in the LC cells with PMMA+DR1 substrates, one can anticipate two possible reasons: **(i.)** the orientation of the DR1 molecules, and **(ii.)** the rigidity of the PMMA matrix.

Reason **(i.)** comes from both theoretical considerations and experimental data, evidencing that rod-like molecules often have a tendency to orient perpendicular to the free (air contacting) surface of the film [2]. Such an orientation of the azo-benzene derivatives is unfavorable for photoalignment when the light irradiation is performed (as in our case) with normal incidence to the film (substrate) plane because this orientation results in poor light absorption. Assumption **(i.)** can be, however, tested by a slantwise illumination [29]. Namely, when illuminated with non-polarized light, the trans azo-benzene derivatives tend to reorient with their long axis in the direction parallel with the light propagation direction. Following the work of Ref. [29] we have tried to influence the initial orientation of DR1 molecules in the substrate made of PMMA1+DR1 mixture. In order to do that, after the preparation of the substrate from PMMA1+DR1 mixture, the polymer layer was illuminated slantwise from a non-polarized λ=(457±7)nm light source with illumination dose of $7.5\;J/{\mathrm{cm}}^{2}$ and with light propagation direction, which encloses $30^{\circ }$ with the polymer film plane. The LC cell was then assembled, and prior as well as during filling the cell with E7, it was again illuminated with the same non-polarized light source in the same geometry with a dose of $7.5\;J/{\mathrm{cm}}^{2}$. Such a procedure is supposed to reorient the long axis of DR1 molecules so that they enclose $30^{\circ }$ with the polymer film plane, making the photoalignment experiments much more efficient. Photoalignment measurements on this LC cell, however, have led to results very similar to those shown in Figure 2 and Figure 3b.

Therefore, we assume that reason **(ii.)**, i.e., the rigidity of the PMMA matrix in the glassy state stays behind the poor photoalignment performance of the polymer layers made from PMMA+DR1 mixtures. Presumably, the rigid matrix hinders the cooperative motion (induced by the trans-cis isomerization of the DR1 molecules) necessary for an efficient photoalignment. In contrast to that, recently we have shown for a polymer segment of pDR1 that the trans-isomer of the azo-benzene moiety can take any direction at an energy expense of few RT, more likely due to the flexibility of the main chain than to the flexibility of the short spacer that connects the azo-dye with the polymer chain [41].

The results obtained from AFM scans on polymer reliefs in contact with the air are in line with the photoalignment measurements. The pDR1 surface evidently becomes smoother after the illumination, and the photoinduced changes in surface relief are accompanied by a significant photoinduced mass transfer. In contrast, the relief of the PMMA1+DR1 surface does not change noticeably upon the illumination, and the photoinduced mass transfer was found very close to the resolution of the AFM.

## 5. Conclusions

The results of the specific system presented in this work give a definite answer to the question posed in the title: the photoalignment is far more efficient when the azo-dye DR1 is chemically attached to the PMMA backbone, compared to the case when PMMA and DR1 are physically mixed. We attribute the poor photoalignment performance of the polymer layers prepared from PMMA+DR1 mixtures to the rigidity of the PMMA matrix.

The long-lasting (at least for hours, which follows from the timetable of the AFM measurements) photoinduced changes in the surface relief of pDR1, accompanied with significant photoinduced mass transfer support most of the results on photoalignment measurements at temperatures close to the room temperature. Namely, when in those measurements liquid crystals with phenylcyclohexane or bicyclohexane rigid core were contacting the pDR1 layer, extremely slow, or no back-relaxation occurred upon switching off the pump beam [40,41]. On the other hand, when LCs having biphenyl rigid core interface the pDR1 layer, the mechanisms of the fast back-relaxation shown in Figure 2 and reported in [39], as well as of the zenithal photoalignment at high temperatures shown in Figure 3a and in [39] still remain somewhat puzzling. AFM scans made at the pDR1 surface in contact with the air can not give insights into those mechanisms, presumably due to the absence of π-π aromatic interaction between the contacting medium (air) and the azo-benzene of pDR1.

## Data Availability

The data presented in this study are available on request from the corresponding author.
